# Supplementary material for: Anterior and posterior ratio of corneal surface areas: A novel index for detecting early stage keratoconus
Source: PLoS One. 2020 Apr 2;15(4):e0231074. doi: 10.1371/journal.pone.0231074 (PMC7117727; doi:10.1371/journal.pone.0231074)
Supplement: Attachment — Submitted filename: revise.pdf [file pone.0231074.s002.pdf]

There are some important points I would like to address:

Methods: Lines 126- 133

1. Explain KCN and FFKCN classification methods more clearly. For example, how much skewed-axis was included in the KCN group? Same for other group classifications.

Analysis:

2. The important point in this paper is this study does not evaluate the sensitivity and specificity of the As/Ps index compared to other indices. If you want to say that this index is better than the others, a specific analysis should be performed to compare the area under the curves (AUCs). The area under the curve alone cannot confirm As/Ps ratio has a high diagnostic ability for early KCN detection. It is recommended to use the "DeLong" method to compare the area under the curves and report sensitivity, specificity and optimal cut points. In retrospective data reporting these diagnostic indices also possible.

Results:

3. Line 117, you said keratosis was a reference for the analysis. Why did you compare FFKCN with the control group in Table 1?
4. You should remove the KCN column from the table or report that the p-value related to the comparison of KCN with the normal group.

Discussion:

5. Explain in the discussion why the AUC of As had weak value however, the anterior (As) and posterior (Ps) ratio had high AUC value. (based on Table 2)
6. Curvature indices (e.g ISValue) and new combined indices (e.g BAD-D) appear to had better diagnostic accuracy for early KCN detection. Why this new **elevation based** index (As/Ps) had better diagnostic power than curvature indices OR new combined indices to detect early stages of the disease?
