# Supplementary material for: Anterior and posterior ratio of corneal surface areas: A novel index for detecting early stage keratoconus
Source: PLoS One. 2020 Apr 2;15(4):e0231074. doi: 10.1371/journal.pone.0231074 (PMC7117727; doi:10.1371/journal.pone.0231074)
Supplement: Attachment — Submitted filename: Comment.docx [file pone.0231074.s004.docx]

Comment 2 (analysis section) not been addressed correctly. You can select the optimal cut-off points based on lowest difference between specificity and sensitivity for your cases. (With SPSS or STATA software) The "larger AUC" is not correct for comparing the variables (in the abstract and text).

Table 2 should be revised accordingly.
