# Supplementary material for: Anterior and posterior ratio of corneal surface areas: A novel index for detecting early stage keratoconus
Source: PLoS One. 2020 Apr 2;15(4):e0231074. doi: 10.1371/journal.pone.0231074 (PMC7117727; doi:10.1371/journal.pone.0231074)
Supplement: Attachment — Submitted filename: RESPONSE TO REVIWER3.docx [file pone.0231074.s005.docx]

RESPONSE TO REVIWER3

We wish to express our appreciation to the reviewer for careful reading our manuscript and for insightful comments. The comments have helped us significantly improve manuscript “Anterior and Posterior Ratio of Corneal Surface Areas: A Novel Index for Detecting Early Stage Keratoconus”. We have revised the manuscript according to the various suggestions made by the reviewer as below.

1. The authors have revised the majority of the manuscript according to the reviewers' suggestions. However, the mini-data set is still not available for further review

Response to Comment 1: We greatly appreciate the Reviewer’s very insightful and helpful comment. We have now added the data set which include the values to use the ROC analysis as supporting information (S1 dataset). We believe that this new information adequately addresses the reviewer's comment.
